# 306. Patient-Derived Airway Organoids: a Pre-clinical Platform to Model RSV Infection

**DOI:** 10.1093/ofid/ofae631.096

**Published:** 2025-01-29

**Authors:** Alessandra Merenda, Nilofar Ehsani, Foteini Gkogkou, Johanna Pott, Taya Bezhaeva, Robert G J Vries, Sylvia F Boj

**Affiliations:** HUB Organoids B.V., Utrecht, Utrecht, Netherlands; HUB Organoids, Utrecht, Utrecht, Netherlands; HUB Organoids, Utrecht, Utrecht, Netherlands; HUB Organoids, Utrecht, Utrecht, Netherlands; HUB Organoids B.V., Utrecht, Utrecht, Netherlands; HUB Organoids B.V., Utrecht, Utrecht, Netherlands; HUB Organoids B.V., Utrecht, Utrecht, Netherlands

## Abstract

**Background:**

HUB’s patient-derived organoids (PDOs) are novel “mini-organs in a dish” derived from adult epithelial stem cells which form 3D structures and resemble the architecture and physiology of the tissue of origin. Airway PDOs represent a valuable model for investigating the pathogenesis of respiratory infections and testing strategies to prevent or treat such diseases. Because *in vivo* pathogens attack the luminal surface of the epithelium, HUB has established monolayers from lung PDOs, where the luminal side is accessible for mimicking host-pathogen interactions.

Viruses such as the Respiratory Syncytial Virus (RSV) are associated with severe respiratory illnesses, especially in children and older adults. RSV uses the fusion protein, F protein, which can interact with the intercellular adhesion molecule-1 (ICAM-1) to facilitate cell entry, rendering both molecules appealing targets for inhibiting RSV infection.

**Methods:**

This study assessed the effect of Rilematovir, an RSV antiviral agent that inhibits the viral F protein and prevents cell fusion in RSV-infected PDOs. To this end, airway PDO-derived monolayers were pre-treated with different concentrations of the inhibitor before infection with RSV. Transepithelial electrical resistance (TEER) and monolayer integrity were monitored daily, and the viral titre was quantified by real-time quantitative PCR (RT-qPCR) at different time points post-infection (PI). The production of IP-10 and CXCL2, two inflammatory cytokines released upon infection, was also measured 7 days PI.

**Results:**

Our data revealed that when no inhibitor treatment was applied, the viral titre increased gradually, indicating that RSV could infect and propagate in airway PDO monolayers. Notably, monolayers treated with F protein inhibitors showed a significant decrease in viral titre. This viral titre decrease was accompanied by a total reduction of IP-10 and CXCL2, suggesting a potential role for F-protein inhibition in modulating RSV infection.

**Conclusion:**

Overall, this study confirms that airway PDO-derived monolayers are innovative models to study viral infections and investigate potential targets to prevent or treat such infections. These findings could potentially lead to significant advancement in the field of respiratory infections.

**Disclosures:**

**All Authors**: No reported disclosures

